# An Atypical Cutaneous Reaction to Rivastigmine Transdermal Patch

**DOI:** 10.1155/2011/752098

**Published:** 2011-01-11

**Authors:** T. Grieco, M. Rossi, V. Faina, I. De Marco, P. Pigatto, S. Calvieri

**Affiliations:** ^1^Department of Dermatology, Universitá di Roma “La Sapienza”, Viale del Policlinico, 155, 00161 Rome, Italy; ^2^Department of Technology for Health, Dermatological Clinic, IRCCS Galeazzi Hospital, University of Milan, 20122 Milan, Italy

## Abstract

Rivastigmine is a cholinesterase inhibitor which improves cognitive function and is currently being used in patients with mild to moderate Parkinson's and Alzheimer's dementia. This drug can be given orally or topically, as transdermal patch. The latter form is currently used for most excellent compliance and few side effects. The most common cutaneous side effects are irritative dermatitis. We report the second case of active sensitization by the rivastigmine-patch in a patient suffering from Alzheimer's dementia.

## 1. Introduction

Rivastigmine is a cholinesterase inhibitor which improves cognitive function and is currently being used in patients with Parkinson's and Alzheimer's dementia. The rivastigmine transdermal patch has been approved since July 2007, and it is now preferred to the oral administration [1]. Safety and tolerability of the rivastigmine were evaluated in several randomised and multicentre clinical trials. We report a second case of active rivastigmine-patch sensitization in a patient suffering from Alzheimer's dementia.

## 2. Case Report

**Figure 1:**
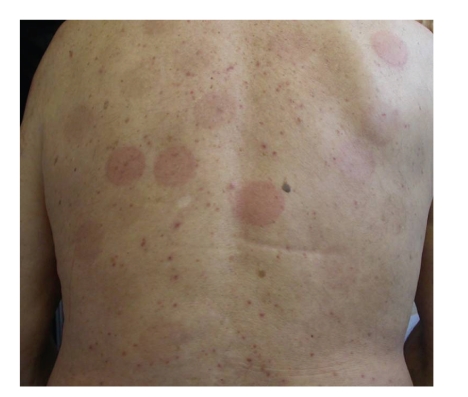
Erythematosus and oedematous patched lesions, bright red, infiltrated, and round in shape on the trunk surface.

In March 2010, a 75-year-old male patient came to our department of Dermatology presenting on the trunk surface and proximal upper limbs, erythematosus, and oedematous patched lesions, sometimes vesicular, bright red, infiltrated, and round in shape (Figure 1). 

The patient, suffering from Alzheimer's dementia, was treated for about a month with rivastigmine 9.5 mg/24 h patch. The skin lesions appeared after about 15 days from the first application of the patch; then, he proceeding with topical corticosteroids, there was only partial skin lesions improvement. When the rivastigmine transdermal patch was applied, recurrent lesions in previous application skin sites are observed. 

The rivastigmine transdermal application was stopped when the patient came to our evaluation, and one month after skin lesions full remission we started the allergological study with European standard series and we added supplementary series for rubbers and resins. All the tests were negative at 72 and 96 hours. 

Therefore, rivastigmine patch was performed with positive result after 48 and 72 hours; in contrast, placebo patch test with support but without drug showed negative result. Three months after, we repeated the tests, confirming the previous results (control test).

Under anesthesiological surveillance, we administered orally rivastigmine at escalating doses from 1.5 mg daily, but after 72 hours from the first administration, the skin lesions reappeared, an event which could be supposed as an “oral trigger proof.”

## 3. Discussion

Rivastigmine is currently used for the symptomatic treatment of Parkinson's and Alzheimer's dementia. This drug can be given orally or topically, as transdermal patch. 

Rivastigmine is usually well tolerated [2], and its side effects are dose dependent, with a frequency of 9.6% in patients who received 9.5 mg/24 h rivastigmine patch, of 8.6% in patients who received 17.4 mg/24 h rivastigmine patch, and 8.1% in those treated with oral formulation [3].

The transdermal form is a once-daily AD treatment. It is recommended to have a single application every 24 hours on clean, dry, hairless, and healthy skin. It should be applied on the upper or lower back, chest, or upper arms. The patch should be removed gently and replaced with a new one every 24 hours. It's recommended not to apply the rivastigmine patch to the same skin area during the next 2 weeks.

The most frequent side effects reported in the literature [4] include gastrointestinal disorders followed by central nervous system disorders and cardiovascular alterations.

Irritant contact dermatitis (90%–98% of cases) are the cutaneous side effects reported during rivastigmine patch applications [5]. They appeared within 12 hours from the application, are characterized by erythematosus, oedematous, and itchy lesions, limited to the area covered by the patch, which usually disappear after a short period of time (24 hours), and are characterized by the absence of relapse. Recently Makris reported the first case of rivastigmine's transdermal patch hypersensitivity [6].

In our patient, in addition to the characteristic clinical manifestations “A POIS man,” we describe an hypersensitization to rivastigmine for three reasons: the occurrence of an adverse reaction after 15 days from the application of the transdermal drug, enough time for a possible specific immunological sensitization, the simultaneous reactivation of more injuries during the application, and finally the adverse reactions persist beyond the 24 hours reported in the literature [7]. Moreover, the simultaneous reappearance of the generalized skin lesions on rivastigmine application areas after oral treatment showed a condition of specific sensitization in our patient. 

The allergological study conducted with rivastigmine positive patch test and the placebo's negative results confirmed the hypothesis of a specific hypersensitivity to the rivastigmine patch in our patient. 

In this case, we recommend to interrupt the topical or systemic therapy, though oral rivastigmine desensitization was recently reported as effective [6].

## References

[1] Sadowsky CH, Dengiz A, Olin JT, Koumaras B, Meng X, Brannan S (2009). Switching from donepezil tablets to rivastigmine transdermal patch in Alzheimer’s disease. *American Journal of Alzheimer’s Disease and other Dementias*.

[2] Winblad B, Cummings J, Andreasen N (2007). A six-month double-blind, randomized, placebo-controlled study of a transdermal patch in Alzheimer’s disease’rivastigmine patch versus capsule. *International Journal of Geriatric Psychiatry*.

[3] Grossberg GT, Sadowsky C, Olin JT (2010). Rivastigmine transdermal system for the treatment of mild to moderate Alzheimer’s disease. *International Journal of Clinical Practice*.

[4] Alva G, Cummings JL (2008). Relative tolerability of Alzheimer’s disease treatments. *Psychiatry*.

[5] Kurz A, Farlow M, Lefèvre G (2009). Pharmacokinetics of a novel transdermal rivastigmine patch for the treatment of Alzheimer’s disease: a review. *International Journal of Clinical Practice*.

[6] Makris M, Koulouris S, Koti I, Aggelides X, Kalogeromitros D (2010). Maculopapular eruption to rivastigmine’s transdermal patch application and successful oral desensitization. *Allergy*.

[7] Lefèvre G, Sedek G, Huang H-LA (2007). Pharmacokinetics of a rivastigmine transdermal patch formulation in healthy volunteers: relative effects of body site application. *Journal of Clinical Pharmacology*.

